# Metagenomic analysis of the vaginal microbiota in cows with ovarian cysts

**DOI:** 10.2478/jvetres-2026-0028

**Published:** 2026-05-28

**Authors:** Gamze Evkuran Dal, Baran Çelik, Ahmet Sabuncu, Merve Yılmaz, Ayşe Ilgın Kekeç, Emek Dümen, Serkan İkiz, Kadir Serdar Diker

**Affiliations:** 1 Department of Obstetrics and Gynecology, Department of Food Hygiene and Technology, Faculty of Veterinary Medicine, Istanbul University-Cerrahpaşa, 34320 Avcilar, Türkiye; 2 Department of Microbiology, Istanbul University-Cerrahpaşa, Faculty of Veterinary Medicine, 34500 Buyukcekmece, Türkiye; 3 Department of Food Hygiene and Technology, Faculty of Veterinary Medicine, Istanbul University-Cerrahpaşa, 34320 Avcilar, Türkiye; 4 Department of Microbiology, Aydin Adnan Menderes University, Faculty of Veterinary Medicine, 09020 Aydın, Türkiye

**Keywords:** bovine ovarian cysts, dairy cattle, long-read 16S rRNA sequencing, postpartum reproductive function, vaginal microbiome

## Abstract

**Introduction:**

This study compared the vaginal microbiota composition of dairy cows with follicular and luteal ovarian cysts using metagenomic analysis.

**Material and Methods:**

Ovarian cysts, which impair reproductive performance through endocrine disruption, were diagnosed by ultrasonography and serum hormone evaluation in Holstein cows 30–60 d postpartum. Forty-five cows were initially included and divided into follicular cyst, luteal cyst and control groups. Vaginal lavage samples were analysed using third-generation sequencing, and taxonomic classification was performed through 16S rRNA gene analysis.

**Results:**

A total of 258 operational taxonomic units (OTUs) were identified, with the highest diversity observed in the control group (mean of 56.8 OTUs) and the lowest in the luteal cyst group (mean of 49.0 OTUs). Proteobacteria was the dominant phylum across all groups (93.4%), followed by Tenericutes (5.9%). Firmicutes, Bacteroidetes and Fusobacteria accounted for less than 1%. At the family level, Burkholderiaceae (62.7%) and Pasteurellaceae (24.0%) were predominant, while of the genera, *Ralstonia* was the most abundant (62.2%). The luteal group had the highest relative abundance of Burkholderiaceae, whereas Pasteurellaceae was most abundant in the control group.

**Conclusion:**

These results indicate that cystic cows exhibit reduced microbial diversity and altered bacterial composition in comparison with healthy animals. The predominance of Proteobacteria and *Ralstonia* suggests a potential link between endocrine imbalance and changes in the vaginal microenvironment. Hormonal analyses supported the classification of cyst types, with follicular cyst cows showing low progesterone (0.31 ± 0.05 ng/mL) and high oestradiol-17β concentrations (55.57 ± 7.91 pg/mL), whereas luteal cyst cows exhibited higher progesterone (2.89 ± 0.74 ng/mL) and lower oestradiol-17β concentrations (6.19 ± 0.56 pg/mL) (P < 0.001). These results may support future studies evaluating vaginal microbial profiles as complementary indicators of ovarian status in dairy cows.

## Introduction

Ovarian cysts constitute a significant pathological condition leading to impaired reproductive performance in dairy cattle. These cystic structures arise as a result of endocrine imbalances within the hypothalamic–ypophyseal–gonadal axis, leading to the persistence of a preovulatory follicle without ovulation (4). The reported incidence in dairy herds ranges from 6% to 19%, making cysts one of the principal contributors to infertility (14).

The occurrence of ovarian cysts is closely linked to the physiological and endocrine adjustments occurring during the postpartum period. In cows, uterine involution is generally complete within approximately 30 to 40 d postpartum. Lochial discharge ceases between days 14 and 18, while cervical involution is typically finalised by around day 30. The endometrium attains full functional readiness for a subsequent pregnancy by approximately 60 d after calving (26). This period of uterine recovery coincides with profound endocrine fluctuations and renewed ovarian activity, conditions that may predispose cows to ovarian dysfunction. Accordingly, although ovarian cysts may occur at various stages of lactation, they are most frequently identified within the first 60 d postpartum (40).

Follicular cysts are defined as ovarian structures measuring at least 2.5 cm in diameter that persist for more than ten days in the absence of a corpus luteum. Luteal cysts have a similar morphology but differ in wall thickness. In follicular cysts, progesterone concentrations remain low, whereas oestradiol levels are generally elevated, the extent of elevation depending on the cyst subtype. By contrast, luteal cysts are characterised by elevated progesterone secretion. Diagnosis relies on a combination of clinical observations, such as behavioural changes, and findings from rectal palpation, steroid hormone assays and transrectal ultrasonography (14, 21). In ultrasonographic evaluations, follicular cysts typically exhibit a wall thickness of ≤3 mm with anechoic contents, whereas luteal cysts have a wall thickness >3 mm and contain echogenic particles and network-like patterns (40). Additionally, non-steroidogenic cysts may be encountered; these hormonally inactive structures do not alter the oestrous cycle and can coexist with a functional corpus luteum (38, 40).

While the endocrine and morphological characteristics of ovarian cysts are well described, less attention has been paid to the potential role of the reproductive tract microenvironment in their development. Studies investigating the role of vaginal microflora in maintaining reproductive tract health in women have highlighted the significance of preventing infections caused by dominant microorganisms and the benefits of targeted treatments; these findings have inspired parallel research in veterinary medicine (37, 41). A review of the literature reveals that, although several investigations have examined the association between vaginal flora and factors such as genital tract health, pregnancy status and age in cows – using both culture-based methods and metagenomic analyses – no studies have specifically addressed its relationship with ovarian cysts. Metagenomic approaches enable the identification of unculturable bacteria through ribosomal RNA gene sequencing, thereby allowing the characterisation of microbial populations that contribute to the homeostasis of specific tissues (32). In contrast, culture-based methods can only detect a fraction of the microbial community present, leaving many species undetected (37, 41).

In the human vaginal microbiota, *Lactobacilli* play a dominant role by maintaining vaginal pH below 4.5 through lactic acid production, thereby inhibiting numerous pathogenic bacteria. In cows with a healthy genital tract, *Lactobacillus* species have also been detected, although not as abundantly as in humans. Vaginal pH in cattle has often been reported as near neutral (9). In healthy cows, lactic acid bacteria can be dominant and exhibit probiotic properties, whereas in cows with endometritis, bacterial diversity is notably higher (41).

Culture-based studies have shown varying microbial profiles. Otero *et al*. (24) reported *Enterococci* and *Staphylococci* as the dominant genera, and *Enterobacteriaceae* and *Lactobacilli* following them. Additionally, *Pediococcus*, a genus closely related to *Lactobacillus* and containing bacteria capable of producing antimicrobial compounds, was identified in the vaginal microbiota of cows. Wang *et al*. (42) found that, rather than originating from a stable genital tract flora, infections may originate from environmental sources (*e.g. Bacillus* spp.), skin (*Staphylococcus* spp.) or faecal material (*E. coli*, lactic acid bacteria).

The essential role of commensal bacteria in the development of the mucosal immune system has been demonstrated in germ-free animal models using gnotobiological techniques. Disruptions in these microbial communities have been implicated in complex multifactorial diseases such as inflammatory bowel disease, periodontal disease, rheumatoid arthritis, atherosclerosis, allergies, multiple organ failure and colorectal cancer. This association is linked to potent immuno-activating components of the microflora, including lipopolysaccharides, peptidoglycans, superantigens, bacterial DNA and heat-shock proteins (39).

Studies comparing the vaginal microbiota of cows have shown that even minor shifts in microbial composition can transform commensal–host interactions into pathogenic ones. In the healthy vaginal ecosystem, anaerobic microorganisms are predominant, whereas during pathological conditions, the abundance of aerobic and facultative anaerobic bacteria increases. Acidic metabolites produced by certain microorganisms can modify the vaginal pH, indirectly creating favourable conditions for the proliferation of other bacterial species and promoting the formation of new micro-ecosystems. Therefore, the composition of the bovine vaginal microbiota is determined by a combination of host-dependent and host-independent factors and is closely associated with vaginal health status (32).

Research examining the relationship between pregnancy, age and vaginal flora has indicated that individual variation exerts a greater influence than these factors alone. Nevertheless, non-pregnant cows have been reported to possess a more diverse and abundant bacterial community. This reduction in microbial load during pregnancy is consistent with observations that the elevated bacterial population detected at oestrus tends to decline under the influence of increased progesterone secretion (16).

The objective of this study was to compare the vaginal microbiota of dairy cows with follicular and luteal ovarian cysts, as well as clinically normal cows, during the late postpartum period using a metagenomic approach. Vaginal samples were analysed to characterise microbial distribution patterns among the study groups.

## Material and Methods

### Sampling

Forty-five Holstein cows were included in the study. Animals were randomly selected from dairy farms in Istanbul province that complied with hygiene and sanitation standards. The study population consisted of both primiparous and multiparous cows, aged 2–5 years, that were assessed as clinically healthy based on general, gynecological and udder examinations. Only cows with a body condition score of 3–4, between 30–60 d postpartum and with no history of antibiotic treatment within the previous two months were enrolled.

### Preliminary grouping

Based on transrectal ultrasonographic examination, three preliminary groups were formed, each consisting of 15 cows. Animals with ovarian cysts characterised by a wall thickness of ≤3 mm and anechoic content were assigned to the follicular cyst group (n = 15), whereas those with cysts having a wall thickness >3 mm and echogenic content were assigned to the luteal cyst group (n = 15) (6). Non-pregnant cows in the postpartum period (30–60 d) with no pathological ovarian structures and showing cyclic ovarian activity were included in the control group. To ensure homogeneity, only dioestrous cows with a mature corpus luteum were selected for the control group. All ultrasonographic examinations were performed using a B-mode ultrasound device equipped with a 5 MHz linear probe (MyLab Five Vet; Esaote Pie Medical, Genova, Italy).

### Collection of samples

Vaginal lavage samples were collected from all cows under aseptic conditions immediately after ultrasonographic examination. A flexible Foley catheter was inserted, and 50 mL of sterile distilled water was infused intravaginally. The fluid was aspirated with a sterile 50 mL syringe and transferred into Falcon tubes. Blood samples were simultaneously collected from the jugular vein into anticoagulant-free tubes. Vaginal lavage and blood samples were transported to the laboratory under controlled temperature conditions to preserve sample integrity. Vaginal lavage specimens were transferred using vapour-phase liquid-nitrogen shippers ensuring cryogenic stability, while blood samples were kept under refrigerated cold-chain conditions during transport. Blood samples were centrifuged at 2,000 rpm for 10 min (NF 800R; Nüve, Ankara, Türkiye), and the separated serum was transferred into 1.5 mL Eppendorf tubes and stored at -20°C until analysis. Vaginal lavage samples were stored in an ultra-low temperature freezer (-80°C) until DNA extraction and metagenomic sequencing were conducted (16).

### Definitive classification of groups

**Fig 1.**
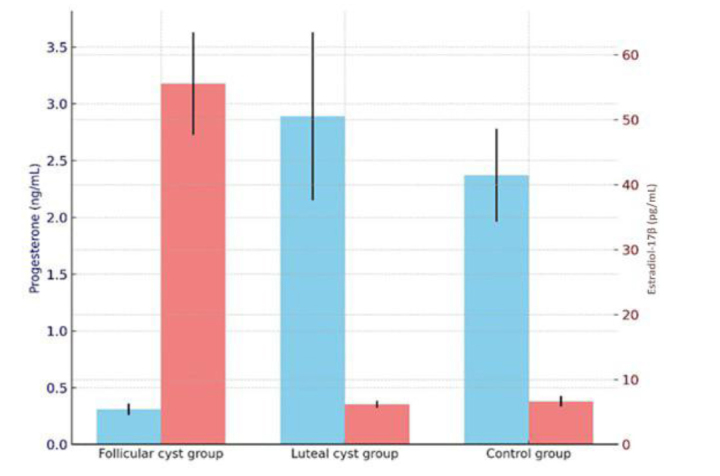
The mean serum hormone concentrations in the vaginal lavage samples of primiparous and multiparous Holstein dairy cows

Serum I7β-oestradiol and progesterone concentrations were measured using commercial ELISA kits according to the manufacturers’ instructions (Bovine Estradiol and Progesterone ELISA Kits from BT-Lab, Shanghai, China; a Robonik ELISA Reader and Washer from Navi, Mumbai, India; and an NE500 Incubator from Nüve). Five animals were excluded from the study because they were culled by farm management for health reasons. After integrating the ultrasonographic findings with serum hormone profiles (Fig. 1) as described by Karsavuranoğlu *et al*. (13), the final grouping of the animals was established as follows: a follicular cyst group with 14 cows, a luteal cyst group with 13 and a control group also with 13 animals.

The differences among three groups were analysed using one-way ANOVA. After obtaining a significant result, Duncan’s multiple-comparison test was used to determine which groups differed. A significance level of P-value < 0.05 was considered statistically significant.

### Metagenomic analysis: DNA extraction

The commercial kit protocol was followed for the bacterial DNA extraction procedure (MagAttract HMW DNA Kit; Qiagen, Hilden, Germany). For the microbiome analyses, the density of DNA samples were determined by Qubit 2.0 fluorimeter (ThermoFisher Scientific/Invitrogen, Carlsbad, CA, USA) after the DNA extraction procedures. The amounts of DNA in the samples were recorded. Extracted DNA samples were stored at -20°C until the microbiome analyses were performed.

### Metagenomic analysis: microbiome analyses

After DNA density values were determined the densities of DNA samples were regulated following the protocol of the Oxford Nanopore rapid sequencing 16S Barcoding Kit (cat. No. SQK 16S024; Oxford Nanopore Technologies, Oxford, UK). Finally barcoding and PCR procedures were performed as described below (Table 1 and 2).

**Table 1.** PCR mixture constituents for cystic and healthy primiparous and multiparous Holstein dairy cow vaginal microflora analyses

LongAmp Taq 2x Master Mix	25 μL
Barcode Primer	10 μL
double-distilled H_2_O	5 μL
Template DNA	10 μL
Total volume	50 μL

**Table 2.** PCR thermal cycling for cystic and healthy primiparous and multiparous Holstein dairy cow vaginal microflora analyses

Temperature	Duration	Number of cycles
95°C	1 min	1
95°C	20 s	
55°C	30 s	25
65°C	2 min	
65°C	5 min	1
4°C	∞	1

The PCR products were purified by AMPure XP (Beckman Coulter, Brea, CA, USA). Barcoded DNA pools were generated by gathering all the samples into an Eppendorf tube after the measurement of the densities of the purified products. One μL of RAP, the rapid sequencing adapter reagent (Oxford Nanopore) was added to the pool for binding the adaptors and the mixture was incubated for 5 min. The product was purified by AMPure after the adaptors bound. Then the loading buffer was formed as the protocol instructed. A Flow Cell Priming Kit (Oxford Nanopore Technologies) was used in the loading procedure with cells which had R9 chemistry, and the procedure was completed as indicated in the protocol. After the loading procedure was completed, the products were transferred to a Flow Cell MinION device (Oxford Nanopore Technologies), and a 24-h sequencing protocol was performed using the MinKNOW program (Oxford Nanopore Technologies). Bioinformatic analysis was conducted on the obtained data. After the completion of sequencing, the data were transformed from fast5 to fastq by using guppy v3.1.5 software (basecalling and multiplexing) within MinKNOW. Barcode and adaptor sequences were cleaned by using Porechop v. 0.2.3 (44). In addition, 45 base pairs were deleted from both edges of the sequences to remove universal primers and labels. After cleaning of the sequences, 1,350–1,550-bp readings were filtered and the rest of the readings were excluded. Cleaned readings were analysed by using the mothur v.1.39.5 specialised workflow analysis platform (36). Sequences were purified from chimeric structures and aligned, and the distances were measured between the sequences and the similarity matrix. The readings that showed 99% and above similarity were aggregated. Thus, operational taxonomic units (OTUs) were generated. These OTUs were compared according to the Ribosome Database Project (8) 16S rRNA database and taxonomical annotations were formed. Statistical results were obtained by correlating the same sorted OTUs. The graphics were generated using Minitab Statistical Software (Minitab, State College, PA, USA) and R (29).

### Statistical evaluation

The differences between the three groups in serum hormone concentrations were analysed using one-way ANOVA. After obtaining a significant result, Duncan’s multiple-comparison test was used to determine which groups differed. A significance level of P-value < 0.05 was considered statistically significant. Metagenomic differences were found by subjecting data to a Kruskal–Wallis test. The SPSS v. 13.0 program (SPSS, Chicago, IL, USA) was used for comparing the statistical differences among the groups.

## Results

The analysis of serum hormone concentrations revealed distinct endocrine patterns between follicular and luteal cystic conditions. The mean serum progesterone concentrations were 0.31 ± 0.05 ng/mL in cows with follicular cysts (P-value < 0.001), 2.89 ± 0.74 ng/mL in those with luteal cysts and 2.37 ± 0.41 ng/mL in the control group. The mean I7β-oestradiol concentrations were 55.57 ± 7.91 pg/mL (P-value < 0.001), 6.19 ± 0.56 pg/mL and 6.64 ± 0.81 pg/mL for the follicular cyst, luteal cyst and control groups, respectively. Cows with follicular cysts exhibited persistently low progesterone and elevated 17β-oestradiol levels, reflecting the sustained oestrogenic activity and absence of luteal tissue. In contrast, luteal cyst cases showed markedly higher progesterone concentrations and suppressed 17β-oestradiol levels, consistent with partial luteinisation of the follicular wall and reduced follicular steroidogenic activity. These hormonal differences confirm the distinct pathophysiological nature of follicular and luteal cysts in cattle (5, 9, 13).

The metagenomic analyses determined 258 OTUs. In the control group, the highest OTU count was 85 (in sample (S_) 36) and the lowest was 34 (in S_31 and S_41), the mean being 56.8. In the luteal group, the highest OTU count was 77 (S_015), and the lowest was 38 (S_17). The mean value in this group was 49. Finally in the follicular group, the highest was 109 (S_05), the lowest was 14 (S_06), and the mean was 53.2. Comparing means, the highest OTU count was in the control group, and the lowest was in the luteal group.

**Fig. 2.**
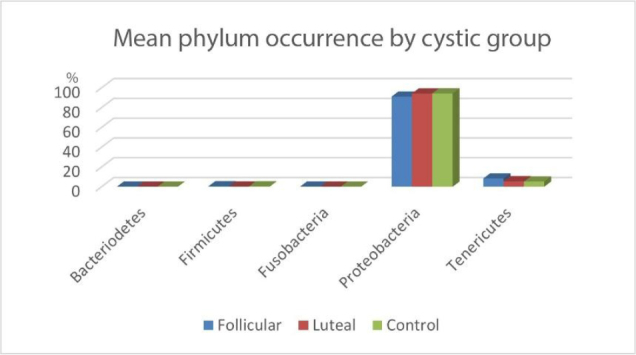
Mean phylum occurrences in cystic and healthy primiparous and multiparous Holstein dairy cow vaginal microflora

When the results were evaluated at the phylum level, Proteobacteria was dominant (93.4%). The control group had the highest mean rate of Proteobacteria occurrence (94.3%), that of the luteal group was nearly identical (94.2%), and the follicular group had the lowest rate (91.1%). The Tenericutes phylum (5.9%) was the second most abundant. This phylum was present in the follicular group at the highest rate (8.2%) and in the control group at the lowest rate (5.2%), which was little different from the luteal group rate. The Firmicutes, Bacteriodetes and Fusobacteria phyla were determined to comprise 0.42%, 0.02% and 0.06% of the detected bacteria, respectively. The follicular group contained the most Firmicutes (0.45%), while the luteal group had the least (0.16%). Hosting of the Bacteriodetes phylum was marginally more evident in the follicular group than in the control group (0.01%), but no Bacteriodetes phylum members were detected in the luteal group. Regarding the Fusobacteria phylum, the highest rate was in the follicular group (0.1%), but the the control group rate was similar. Bacteriodetes phylum was not detected in the luteal group (Fig. 2). When looking at the differences in richness and evenness at the phylum level between groups, the differences were found to be significant, as the P-value was < 0.001 (Fig. 3).

**Fig. 3A:**
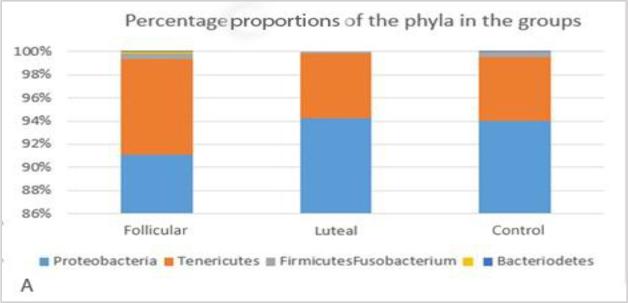
Proportions of the phyla in the groups

**Fig. 3B:**
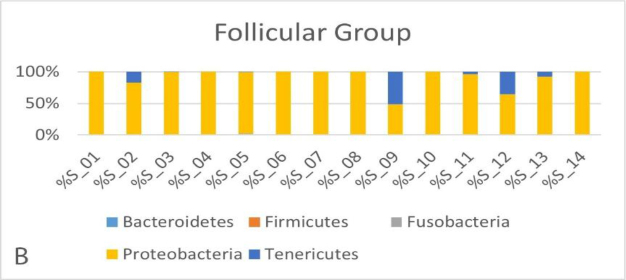
Proportions of the phyla in the follicular cyst group

**Fig. 3C:**
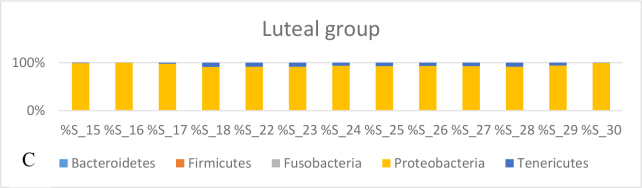
Proportions of the phyla in the luteal cyst group

**Fig. 3D.**
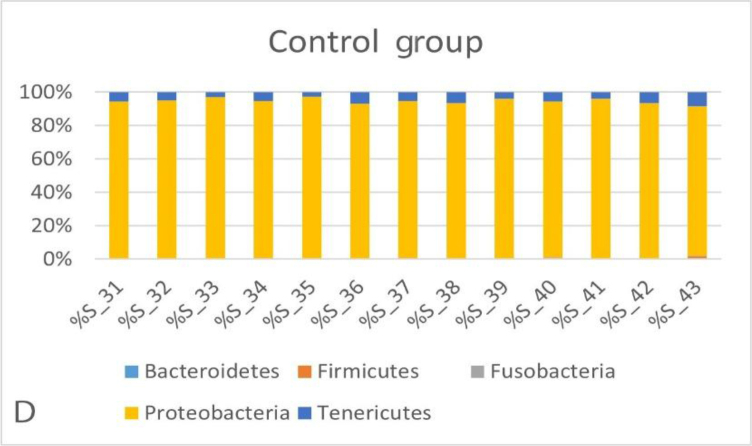
Proportions of the phyla in the control group

**Fig. 4.**
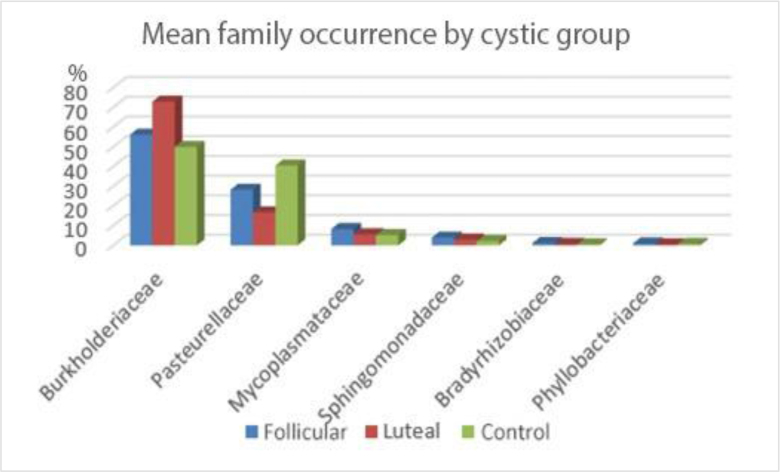
Mean bacterial family occurrence in cystic and healthy primiparous and multiparous Holstein dairy cow vaginal microflora

Burkholderiaceae was the largest proportion in all the groups (62.7%) when the data were examined at family level. The highest rate was observed in the luteal group (72.9%), and the lowest rate was in the control group (49.9%). The second family ranked by occurrence was Pasteurellaceae (24%), and this family was detected at its highest rate in the control group (40.5%). The follicular group followed the control group for carriage of Pasteurellaceae, with a rate of 28.0%, and the luteal group’s percentage was 16.0%. The Mycoplasmataceae family was detected with the highest percentage in the follicular group (8.2%) and was noted at percentages in the luteal and control groups which were lower and similar to each other (respectively 5.5% and 5.2%). Sphingomonadaceae was in fourth place, with a highest percentage of 3.9% in the follicular group. Its detection rates were 2.9% in the luteal group and 2.2% in the control group. The Bradyrhizobiaceae and Phyllobacteriaceae families were approximately 1% of the content in the follicular group and below 1% in both the luteal and the control groups (Fig. 4).

**Fig. 5.**
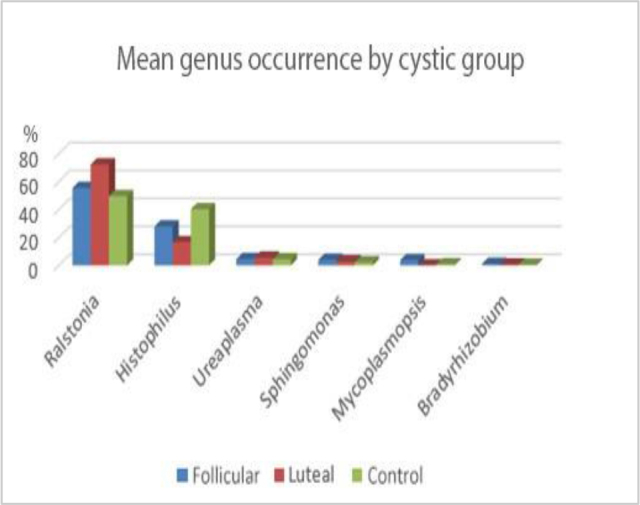
Mean bacterial genus occurrence in cystic and healthy primiparous and multiparous Holstein dairy cow vaginal microflora

**Fig. 6.**
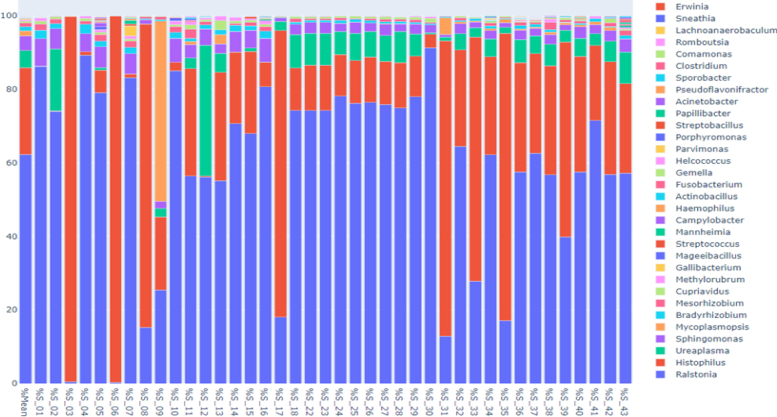
Proportions of the detected genera in cystic and healthy primiparous and multiparous Holstein dairy cow vaginal microflora

*Ralstonia* was the major presence (62.2%) when the results were evaluated to find genera. This genus had the highest rate of prevalence in the luteal group (72.3%). It was observed at 55.4% presence in the follicular group and 49.5% in the control group. The *Histophilus* genus followed *Ralstonia* at 23.6% in this group, but was detected in far lower proportion to *Ralstonia* where it was least present: in the luteal group, where it comprised only 16.6% of bacteria. The prevalence rate of *Histophilus* was noted as 27.8% in the follicular group. The *Ureaplasma* genus’ prevalence was determined as 5.5% in the luteal group, 4.5% in the follicular group, 4.3% in the control group and 4.7% as the mean. The *Mycoplasmopsis* (1.3%) and *Bradyrhizobium* (1.1%) genera were found with the highest rates (3.7% and 1.1%, respectively) in the follicular group, while they were detected at rates below 1% in the luteal and the control groups (Figs 5 and 6).

No significant difference analysis was conducted in the study among the primiparous and multiparous animals because of insufficient animals being available to sample (n = 6).

**Fig.7.**
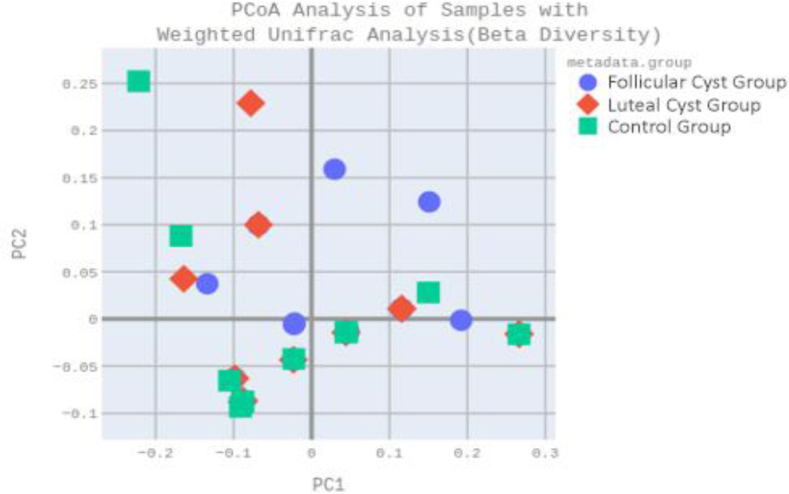
Beta diversities of cystic and healthy primiparous and multiparous Holstein dairy cow vaginal microflora shown through weighted UniFrac distances and principal component analysis (PCoA). PC1/2 – principal component 1/2

Beta diversities were evaluated by using weighted UniFrac distances (19), and sample relationships were visualised using principal coordinates analysis. The control and luteal group diversities were similar, while those of the follicular group were different (Fig. 7).

## Discussion

Ovarian cysts in dairy cattle are known to generate considerable financial losses by extending the interval from calving to successful conception and increasing the need for repeated therapeutic interventions; according to Rudowska *et al*. (34), the economic implications of these disorders heighten the necessity of evidence-based approaches in their management. In this context, microbiota-based assessments offer a valuable perspective, providing insight into reproductive tract homeostasis and helping to identify microbial tendencies that may reflect the differing physiological states of cows with and without cystic conditions, while also providing additional context that can be taken into account when considering available therapeutic approaches.

Microbiota-based assessments provide contextual biological information that may complement clinical and reproductive findings in cows with cystic ovarian conditions. By describing microbial patterns associated with reproductive tract homeostasis and deviation, such analyses can support evidence-based interpretation without implying direct causality. Accordingly, the present study employed a long-read sequencing approach to characterise genital-tract microbial communities with sufficient detail to inform biological discussion. This approach was enabled by third-generation sequencing methods, which generate much longer contiguous reads than second-generation platforms and thereby provide much more comprehensive data (17).

Operational taxonomic unit numbers classify bacteria according to similarities of 16S rRNA marker gene sequences; these were acquired after the reading stage. The OTU counts gave us the number of different units of the microbiota which were gathered from the lower genital system of the sampled cattle in this study. Typically, an OTU set was identified to distinguish the bacteria at genus level with 97% identity of 16S gene sequences (22). The results reflected the diversity of microbiota collected from the lower genital tract of cows with healthy and cystic ovaries. The average OTU count of the control group was calculated as 56.8, that of the follicular cyst group at 53.2, and it was 49.0 in the luteal cyst group. Thus, cows with healthy genital systems exhibited healthier microflora compared with those affected by ovarian cysts. This pattern may have been influenced by hormonal changes associated with the type of ovarian cyst. These hormone–microbiota interactions could play a role in shaping the vaginal environment and may contribute to maintaining reproductive balance.

The vaginal microbiota of cattle has been investigated primarily through culture-based techniques aimed at identifying pathogens responsible for reproductive tract diseases (41). However, with the emergence of advanced metagenomic techniques, cattle vaginal bacterial communities have been identified at the phylum level. Among Nellore, Holstein and Fleckvieh cattle, the major phyla included Firmicutes, Bacteroidetes and Proteobacteria (16, 23). In cows with metritis, the microbial structure was characterised by relatively higher proportions of Bacteroidetes and Fusobacteria, whereas Proteobacteria and Tenericutes were comparatively reduced. These compositional changes corresponded to a loss of bacterial diversity and decreased richness in the uterine microbiota (1, 10). When data from the current study were analysed at the phylum level, Proteobacteria massively dominated all three groups, Tenericutes was a sixteenfold smaller proportion, and Firmicutes, Bacteroidetes and Fusobacteria accounted for less than 1%. The predominance of Proteobacteria may reflect the characteristic microbial composition of the late postpartum period, when the genital tract has largely recovered but may still retain microbial features influenced by recent parturition.

At the family level, Burkholderiaceae was detected to be the most abundant taxon: considerably over half of the identifications were to this family in all three groups. The highest rate was observed in the luteal group and the lowest in the control group. This family is characterised by the presence of ecologically extremely diverse organisms and includes environmental saprophytic organisms, phytopathogens, opportunistic pathogens and primary pathogens for humans and animals (7). An increased abundance of Burkholderiaceae has also been reported in women near term (2). The same family has been detected in the vaginal microbiota of dogs and pigs, as well as in the uterine samples of cows (3, 27, 35). The increased representation of Burkholderiaceae in the luteal group might be related to hormonal influences or to environmental exposure during or after parturition. Similar findings in human and animal studies indicate that this family can transiently colonise mucosal surfaces during hormonally dynamic periods. The second most abundant family taxon was Pasteurellaceae, which was on average less than half as frequent as Burkholderaceae, and it was at its highest proportion in the control group. In a study analysing 302 bovine placenta samples from abortion cases, Pasteurellaceae were detected in 19 specimens (43). Additionally, an association has been reported between Pasteurellaceae and the vaginal microbiota of cows affected by repeat breeder syndrome (12). Mycoplasmataceae was detected to be the third in abundance. These organisms have been linked to uterine diseases in cattle (15). The fourth most abundant family, Sphingomonadaceae, has been identified in bovine semen and was found at significantly higher levels in the endometrial microbiome of cows with endometritis. Members of this family are thought to contribute to the pathogenesis of endometrial inflammation (18).

Regarding bacterial genera, *Ralstonia* was the most dominant genus across all groups in this study. In dogs, *Ralstonia*, along with *Hydrotalea* and *Fusobacterium*, has been described as one of the most common vaginal genera (20). Furthermore, recent studies have reported that *Ralstonia* species are also highly prevalent in milk samples obtained from cows with subclinical mastitis, suggesting their potential role as opportunistic environmental or emerging mastitis-associated bacteria (31, 46). Similarly, Giannattasio-Ferraz *et al*. (11) identified aerotolerant genera such as Aeribacillus and Bacillus as dominant taxa in Gyr and Nellore cattle, supporting the observation that environmentally associated and oxygen-tolerant bacterial genera may constitute an important component of the bovine vaginal microbiota under postpartum conditions.

The genus *Lactobacillus* has consistently been detected at low abundance throughout the oestrous cycle, appearing more prevalent during the follicular phase than in the luteal phase. In a study conducted on heifers, *Lactobacillus* was identified in 37% of follicular samples (7/19) and 10% of luteal samples (2/20), with relative abundances remaining consistently low (0.19–0.04%) (28). Otero *et al*. (25) further reported that *Lactobacillus* reached its lowest abundance during the dioestrous phase, when progesterone concentrations were elevated Moreover, certain human populations (30) and all primates (45) do not exhibit a *Lactobacillus*-dominated vaginal ecosystem, suggesting that *Lactobacillus* predominance is not a universal characteristic of vaginal microbial homeostasis across mammalian species. Similarly, the few culture-based studies conducted in livestock have shown that *Lactobacillus* spp. occur in lower proportions compared with other bacterial genera in both bovine and ovine vaginas (25, 33). The low abundance of *Lactobacillus* species in this study is consistent with these previous reports. Instead, a wider range of bacterial genera seem to support reproductive health in cows with normal ovarian activity, while this diversity appears to decline in animals affected by ovarian cysts. This finding suggests that vaginal microbial homeostasis in cattle is maintained through microbial balance rather than the predominance of a single genus. In this context, metagenomic analysis of the vaginal microbiome may contribute to the identification of microbial patterns accompanying ovarian dysfunction, providing a detailed characterisation of the reproductive tract environment and establishing a reference framework for future studies aimed at understanding the complex interactions between ovarian status and microbial dynamics at the herd level.

While hormonal alterations may partly explain the microbial differences observed in cows with follicular cysts, the similar concentrations of I7β-oestradiol and progesterone in cows with luteal cysts and control cows suggest that additional factors may also contribute to microbiota variation in the luteal cyst group. These factors may include metabolic or physiological conditions associated with the postpartum period, which were not specifically evaluated in the present study and therefore warrant further investigation. It should be considered that ovarian cyst–like structures detected during the early postpartum period may, in some cases, reflect transient ovarian changes associated with postpartum endocrine adaptation rather than persistent pathological conditions. Accordingly, the timing of cyst detection after calving may influence the interpretation of microbiome-related differences among the study groups.

In this study, although *Ralstonia* was identified as the most abundant genus across all groups, its presence likely reflected a temporary adaptation to the postpartum vaginal environment rather than an active infection. The cows included in the study were in good overall health without systemic disease and represented both healthy and cystic ovarian conditions. The predominance of *Ralstonia* may therefore indicate an environmentally derived and opportunistic bacterium adapted to the postpartum reproductive tract. This interpretation is supported by recent reports showing high levels of *Ralstonia* in milk samples from cows with subclinical mastitis, suggesting its possible role as an environmentally derived opportunistic microorganism in dairy herds (31, 46).

## Conclusion

This study demonstrated that the vaginal microbiota of dairy cows differs between animals with ovarian cysts and those with clinically normal ovaries during the late postpartum period. Microbial richness, as reflected by OTU values, was highest in the control group (56.8), next highest in the follicular cyst group (53.2) and lowest in the luteal cyst group (49.0), indicating a relative reduction in microbial diversity in cows affected by ovarian cysts. At the phylum level, *Proteobacteria* predominated across all groups (93.4%), while *Burkholderiaceae* was identified as the most abundant family (62.7%). The genus *Ralstonia* was dominant in all groups, suggesting an environmental adaptation within the postpartum reproductive tract rather than a disease-specific association. Overall, the findings indicate that alterations in vaginal microbial composition and diversity are associated with ovarian cyst status in dairy cows. These differences may be related to hormonal conditions within the genital environment during the postpartum period. However, no causal relationship can be inferred from the present findings. Further studies are needed to better understand the relevance of these associations in postpartum reproductive physiology.

## References

[1] Adnane M., Chapwanya A. (2022). Role of genital tract bacteria in promoting endometrial health in cattle. Microorganisms.

[2] Avershina E., Slangsvold S., Simpson M.R., Storrø O., Johnsen R., Øien T., Rudi K. (2017). Diversity of vaginal microbiota increases by the time of labor onset. Sci Rep.

[3] Becker A.A.M.J., Munden S., McCabe E., Hurley D., Fanning S., Chapwanya A., Butaye P. (2023). The endometrial microbiota—16S rRNA gene sequence signatures in healthy, pregnant and endometritis dairy cows. Vet Sci.

[4] Braw-Tal R., Pen S., Roth Z. (2009). Ovarian cysts in high-yielding dairy cows. Theriogenology.

[5] Brito L.F.C., Palmer C.W. (2004). Cystic ovarian disease in cattle. Large Animal Vet Rounds.

[6] Bruijns B., Tiggelaar R., Gardeniers H. (2018). Massively parallel sequencing techniques for forensics: A review. Electrophoresis.

[7] Coenye T., Rosenberg E., DeLong E.F., Lory S., Stackebrandt E., Thompson F. (2014). The Prokaryotes.

[8] Cole J.R., Wang Q., Fish J.A., Chai B., McGarrell D.M., Sun Y., Brown C.T., Porras-Alfaro A., Kuske C.R., Tiedje J.M. (2014). Ribosomal Database Project: data and tools for high-throughput rRNA analysis. Nucl Acids Res.

[9] Douthwaite R., Dobson H. (2000). Comparison of different methods of diagnosis of cystic ovarian disease in cattle and an assessment of its treatment with a progesterone-releasing intravaginal device. Vet Rec.

[10] Galvão K.N., Bicalho R.C., Jeon S.J. (2019). Symposium review: the uterine microbiome associated with the development of uterine disease in dairy cows. J Dairy Sci.

[11] Giannattasio-Ferraz S., Laguardia-Nascimento M., Gasparini M.R., Leite L.R., Araujo F.M.G., de Matos Salim A.C., de Oliveira A.P., Nicoli J.R., de Oliveira G.C., da Fonseca F.G. (2019). A common vaginal microbiota composition among breeds of *Bos taurus indicus* (Gyr and Nellore). Braz J Microbiol.

[12] Gonzalez Moreno C., Torres Luque A., Galvão K.N., Otero M.C. (2022). Bacterial communities from vagina of dairy healthy heifers and cows with impaired reproductive performance. Res Vet Sci.

[13] Karsavuranoğlu G., Saribay M.K., Gözer A., Bahan O. (2022). Ineklerde ovaryum kistlerinin tanısı, tedavisi ve korunma yöntemleri (Diagnosis, treatment and prevention methods of ovarian cysts in cows – in Turkish). Bahri Dağdaş Hayvancilik Araştirma Dergisi.

[14] Kesler D.J., Garverick H.A. (1982). Ovarian cysts in dairy cattle: a review. J Anim Sci.

[15] Knudsen L.R.V., Karstrup C.C., Pedersen H.G., Angen Ø., Agerholm J.S., Rasmussen E.L., Jensen T.K., Klitgaard K. (2016). An investigation of the microbiota in uterine flush samples and endometrial biopsies from dairy cows during the first 7 weeks postpartum. Theriogenology.

[16] Laguardia-Nascimento M., Branco K.M.G.R., Gasparini M.R., Giannattasio-Ferraz S., Leite L.R., Araujo F.M.G., de Maltos Salim A.C., Nicoli J.R., de Oliviera G.C., Barbosa-Stancioli E.F. (2015). Vaginal microbiome characterization of Nellore cattle using metagenomic analysis. PLoS One.

[17] Lee H., Gurtowski J., Yoo S., Nattestad M., Marcus S., Goodwin S., McCombie W.R., Schatz M.C. (2016). Third-generation sequencing and the future of genomics. BioRxiv.

[18] Li J., Zhu Y., Mi J., Zhao Y., Holyoak G.R., Yi Z., Wu R., Zeng S. (2022). Endometrial and vaginal microbiome in donkeys with and without clinical endometritis. Front Microbiol.

[19] Lozupone C.A., Hamady M., Kelley S.T., Knight R. (2007). Quantitative and qualitative β diversity measures lead to different insights into microbial communities. Appl Environ Microbiol.

[20] Lyman C.C., Holyoak G.R., Meinkoth K., Wieneke X., Chillemi K.A., DeSilva U. (2019). Canine endometrial and vaginal microbiomes reveal distinct and complex ecosystems. PLoS One.

[21] Mueller K. (2008). Cystic ovarian disease in cows – diagnosis and treatment decisions. Livestock.

[22] Müller R., Nebel M. (2021). On the use of sequence-quality information in OTU clustering. Peer J.

[23] Nesengani L.T., Wang J., Yang Y., Yang L., Lu W. (2017). Unravelling vaginal microbial genetic diversity and abundance between Holstein and Fleckvieh cattle. RSC Adv.

[24] Otero C., Saavedra L., Silva de Ruiz C., Wilde O., Holgado A.R., Nader Marcias M.E. (2000). Vaginal bacterial microflora modifications during the growth of healthy cows. Let Appl Microbiol.

[25] Otero C., Silva de Ruiz C., Ibañez R., Wilde O.R., de Ruiz Holgado A.A.P., Nader-Macías M.E. (1999). Lactobacilli and Enterococci isolated from the bovine vagina during the estrous cycle. Anaerobe.

[26] Öcal H., Kalkan C., Semacan A., Kaymaz M., Findik M., Rişvanli A., Kökers A. (2015). Çiftlik Hayvanlarinda Doğum ve Jinekoloji.

[27] Pop R.A., Vasiu I., Meroni G., Martino P.A., Dąbrowski R., Tvarijonaviciute A., Fiţ I.N. (2024). Aerobic vaginal microflora in gestational and non-gestational bitches (*Canis lupus familiaris*). Animals.

[28] Quereda J.J., Barba M., Mocé M.L., Gomis J., Jiménez-Trigos E., García-Muñoz Á., Gómez-Martín Á., González-Torres P., Carbonetto B., García-Roselló E. (2020). Vaginal microbiota changes during estrous cycle in dairy heifers. Front Vet Sci.

[29] R Core Team (2023). R: A language and environment for statistical computing.

[30] Ravel J., Gajer P., Abdo Z., Schneider G.M., Koenig S.S.K., McCulle S.L., Karlebach S., Gorle R., Russel J., Tacket C.O., Brotman R.M., Davis C.C., Ault K., Peralta L., Forney L.J. (2011). Vaginal microbiome of reproductive-age women. Proc Natl Acad Sci U S A.

[31] Rifa’i R., Surjowardojo P., Radiati L.E., Hawa L.C. (2025). Characterization of dairy milk microbiota: implications of *Streptococcus parauberis* for food safety and *Ralstonia pickettii* as a pathogen in subclinical mastitis. CyTA - J Food.

[32] Rodrigues N.F., Kästle J., Coutinho T.J.D., Amorim A.T., Campos G.B., Santos V.M., Marques L.M., Timenetsky J., de Farias S.T. (2015). Qualitative analysis of the vaginal microbiota of healthy cattle and cattle with genital-tract disease. Genet Mol Res.

[33] Rodríguez C., Cofré J.V., Sánchez M., Fernández P., Goggiano G. (2011). Lactobacilli isolated from vaginal vault of dairy and meat cows during progesteronic stage of estrous cycle. Anaerobe.

[34] Rudowska M., Barański W., Socha P., Zduńczyk S., Janowski T. (2015). Treatment of ovarian cysts in dairy cows with simultaneous administration of GnRH and PGF2α has no clear advantage over the use of GnRH alone. J Vet Res.

[35] Sanglard L.P., Schmitz-Esser S., Gray K.A., Linhares D.C., Yeoman C.J., Dekkers J.C., Niederwerder M.C., Serão N.V.L. (2020). Investigating the relationship between vaginal microbiota and host genetics and their impact on immune response and farrowing traits in commercial gilts. J Anim Breed Genet.

[36] Schloss P.D., Westcott S.L., Ryabin T., Hall J.R., Hartmann M., Hollister E.B., Lesniewski R.A., Oakley B.B., Parks D.H., Robinson C.J., Sahl J.W., Stres B., Thallinger G.G., Van Horn D.J., Weber C.F. (2009). Introducing mothur: open-source, platform-independent, community-supported software for describing and comparing microbial communities. Appl Environ Microbiol.

[37] Swartz J.D., Lachman M., Westveer K., O’Neill T., Geary T., Kott R.W., Berardinelli J.G., Hatfield B.G., Thomson J.M., Roberts A., Yeoman C.J. (2014). Characterization of the vaginal microbiata of ewes and cows reveals a unique microbiota with low levels of lactobacilli and near-neutral pH. Front Vet Sci.

[38] Şenünver A., Nak Y., Semacan A., Kaymaz M., Fındık M., Rişvanli A., Köker A. (2015). Çiftlik Hayvanlarinda Doğum ve Jinekoloji.

[39] Tlaskalová-Hogenová H., Štěpánková R., Hudcovic T., Tučková L., Cukrowska B., Lodinová-Žádníková R., Kozáková H., Rossmann P., Bártová J., Sokol D., Funda D.P., Borovská D., Řeháková Z., Šinkora J., Hofman J., Drastich P., Kokešová A. (2004). Commensal bacteria (normal microflora), mucosal immunity and chronic inflammatory and autoimmune diseases. Immunol Let.

[40] Vanholder T., Opsomer G., De Kruif A. (2006). Aetiology and pathogenesis of cystic ovarian follicles in dairy cattle: a review. Reprod Nutr Dev.

[41] Wang J., Sun C., Liu C., Yand Y., Lu W. (2016). Comparison of vaginal microbial community structure in healthy and endometritis dairy cows by PCR-DGGE and real-time PCR. Anaerobe.

[42] Wang Y., Ametaj B.N., Ambrose D.J., Ganzle M.G. (2013). Characterization of the bacterial microbiota of the vagina of dairy cows and isolation of pediocin-producing *Pediococcus acidilactici*. Microbiol.

[43] Ward A.C. (1990). Isolation of Pasteurellaceae from bovine abortions. J Vet Diagn Invest.

[44] Wick R.R. (2017). Porechop: adapter trimmer for Oxford Nanopore reads. Github repository.

[45] Yildirim S., Yeoman C.J., Janga S.C., Thomas S.M., Ho M., Leigh S.R., White B.A., Wilson B.A., Stumpf R.M. (2014). Primate vaginal microbiomes exhibit species-specificity without universal *Lactobacillus* dominance. ISME J.

[46] Zhang J., Liu X., Usman T., Tang Y., Mi S., Li W., Yang M., Yu Y. (2024). Integrated analysis of transcriptome and milk metagenome in subclinical mastitic and healthy cows. Anim Biosci.

